# The effects of time frames on self-report

**DOI:** 10.1371/journal.pone.0201655

**Published:** 2018-08-09

**Authors:** Marta Walentynowicz, Stefan Schneider, Arthur A. Stone

**Affiliations:** 1 Dornsife Center for Self-Report Science, University of Southern California, Los Angeles, California, United States of America; 2 Department of Psychology, University of Southern California, Los Angeles, California, United States of America; University of Essex, UNITED KINGDOM

## Abstract

**Background:**

The degree to which episodic and semantic memory processes contribute to retrospective self-reports have been shown to depend on the length of reporting period. Robinson and Clore (2002) argued that when the amount of accessible detail decreases due to longer reporting periods, an episodic retrieval strategy is abandoned in favor of a semantic retrieval strategy. The current study further examines this shift between retrieval strategies by conceptually replicating the model of Robinson and Clore (2002) for both emotions and symptoms and by attempting to estimate the exact moment of the theorized shift.

**Method:**

A sample of 469 adults reported the extent to which they experienced 8 states (*excited*, *happy*, *calm*, *sad*, *anxious*, *angry*, *pain*, *stress*) over 12 time frames (*right now* to *in general*). A series of curvilinear and piecewise linear multilevel growth models were used to examine the pattern of response times and response levels (i.e., rated intensity on a 1–5 scale) across the different time frames.

**Results:**

Replicating previous results, both response times and response levels increased with longer time frames. In contrast to prior work, no consistent evidence was found for a change in response patterns that would suggest a shift in retrieval strategies (i.e., a flattening or decrease of the slope for longer time frames). The relationship between the time frames and response times/levels was similar for emotions and symptoms.

**Conclusions:**

Although the current study showed a pronounced effect of time frame on response times and response levels, it did not replicate prior work that suggested a shift from episodic to semantic memory as time frame duration increased. This indicates that even for longer time frames individuals might attempt to retrieve episodic information to provide a response. We suggest that studies relying on self-report should use the same well-defined time frames across all self-reported measures.

## Introduction

The past few years have witnessed an upsurge of interest in research on biases affecting retrospective self-report, leading to some distrust in memory-based measures and to a growing preference for methods inquiring about the state of an individual at the present moment [1]. Nevertheless, for practical reasons many self-report questionnaires inquiring about the emotional or somatic state of the person used in both clinical and research contexts are likely to remain retrospective. In those questionnaires, individuals are asked to recall and form an evaluation of their experiences over a predefined time period (e.g., a week, or a month). The recall process necessary for valid retrospective self-reports engages the explicit memory system, in which verbalized memories can be actively and consciously searched, recollected, and described to other people. It encompasses two independent but related systems: the episodic and the semantic memory system [2,3]. The episodic system is responsible for conscious recollection of specific personal events within their defined spatio-temporal context (experience-near knowledge); the semantic system enables the acquisition and preservation of decontextualized general knowledge about objects, situations, and relations. Although both systems are involved in memory recall, the degree of their contribution to retrospective self-reports may differ depending on the features of retrieval, importantly, the length of the reporting period [4]. Given the broad range of time frames used in self-report questionnaires measuring affective states [5], subjective well-being [6], and bodily symptoms [7], it is important to understand how and under which circumstances episodic versus semantic memory systems affect retrospective self-reports.

Robison and Clore [4] proposed a framework to explain the role of episodic and semantic memory in self-reports of emotions. Their model is based on the assumption that when asked to provide a rating, individuals use those sources of information that are most relevant to the current evaluation and still accessible. In this view, ratings of one’s current experience and relatively recent past involve access of episodic knowledge, which is event-specific and situated in a particular time and context. When self-reports cover long time frames (e.g., last month/year), access to episodic details becomes more limited. As a result, episodic retrieval is abandoned in favor of a semantic retrieval strategy, which reflects beliefs individuals have about the self and situation.

To test the assumptions of the model, Robinson and Clore [8] used a judgment task during which they asked participants to evaluate their emotions over a range of time frames and recorded the time participants needed to form each response. The response times increased as time progressed for relatively short time frames (i.e., from now to the last few days), whereas the response times remained constant or decreased as time progressed for relatively longer time frames (i.e., from last few weeks to years). The response levels demonstrated a similar pattern—an initial increase in mean response levels for shorter time frames was followed by flattening of the slope in response levels for longer time frames. The results for both response times and levels were interpreted as support for the model [8]. The monotonic increase observed for the time frames shorter than *the last few weeks* could suggest an episodic retrieval strategy, as longer time frames would presumably require more time to retrieve and summarize the experiences. The increase in intensity ratings with the length of the time frame was also expected, based on the assumption that longer time frames allow for more instances of a given experience to be taken into account when forming an evaluation [5,8]. The lack of increase of both response times and levels for time frames of a few weeks or more were interpreted as indicators of the semantic retrieval strategy. If retrieval is based on semantic knowledge, which comprises beliefs rather than the aggregation of particular instances, then recalling information from semantic memory should require similar amounts of time and result in similar response levels regardless of the (long) time frame used.

The assumption that ratings pertaining to longer time frames are driven to a greater extent by semantic rather than episodic knowledge could have important consequences for clinical assessment, as many of the diagnostic tests (e.g., DSM-V [9]) and patient-reported outcomes [10] rely on relatively long recall periods. First, patients with different beliefs about their mental and somatic health could retrospectively report different levels of affective or bodily experiences, even though those levels in reality (when measured in real-time) could be similar between patients. Second, if questionnaires covering longer time frames are used to measure therapeutic outcomes, then the observed change in patient-reported outcomes could reflect beliefs about change (“I was supposed to feel better after having the treatment”) rather than an actual change. This could have implications for clinical trials, where placebo expectations are thought to influence symptom ratings [11]. Therefore, in order to optimize recall periods for retrospective self-report measures [10,12], it seems necessary to establish which time frames are associated with an increased reliance on belief-based semantic knowledge.

### The present research

The model of Robinson and Clore [4] was tested only in two studies [8,13], both including relatively small samples of university students, which limits the generalizability of findings to the general population. Moreover, when analyzing the relationship between the time frames and emotions, the responses were collapsed over different items that might yield different patterns. Finally, the time frames used in previous studies were relatively broad and vague (e.g., last “few” months), which made it challenging to estimate exactly at which point in time a shift from episodic to semantic retrieval strategies takes place. Consequently, the use of a larger sample drawn from the general population, the use of many well-defined time frames, and a replication of the results for several individual items would enhance confidence in the finding that a shift in retrieval strategies occurs and could elucidate when (i.e., which exact recall period) it occurs.

The current study addresses the abovementioned limitations and contributes to the literature about memory processes in self-report in several ways. First, we examined whether the pattern of retrieval proposed by Robinson and Clore [4] for ratings of emotions is also applicable to somatic symptoms. Previous studies examining the impact of time frames on symptom ratings have shown that longer time frames (e.g., last month, last year) are often associated with higher ratings compared to ratings pertaining to shorter time frames [14–17]. Moreover, retrospective overestimation of experienced symptoms seemed to be related to the beliefs that individuals hold about their symptoms [18]. In consequence, understanding if and when a shift in retrieval strategies happens for the ratings of somatic symptoms would help with optimizing the time frames used in symptom questionnaires. Therefore, the judgment task used in this study included six emotions as well as two prototypical and commonly reported symptoms, pain and stress. By including both emotions and symptoms, we attempted to conceptually replicate earlier findings and to extend them to a previously understudied domain.

Second, we aimed to understand the hypothesized shift from episodic to semantic retrieval strategy with more precision. Self-reports based on different recall strategies may substantially contribute to incomparable research findings. Therefore, it is important to determine precisely which reporting periods involve episodic versus semantic retrieval strategies. Previous research suggested that the longest time frame associated with an episodic retrieval strategy could be the last “few” weeks [8], but due to the study design (i.e., presentation of a limited number of vaguely worded time frames) was unable to specify the moment of the shift with great precision. In the current study, we respond to this limitation by incorporating a greater variety of quantifiable time frames, all of which were based on questionnaires frequently used in symptom research.

#### Response times

According to assumptions of the Robinson and Clore model [4], relatively recent time frames elicit an episodic retrieval strategy; using this strategy should require a person more time to recall and summarize the experiences as the length of the time frame increases. Once this strategy is abandoned in favor of a semantic retrieval strategy for longer time frames, similar amounts of time to respond to a question should be required independent of the time frame used. Accordingly, we hypothesized that participants’ response times will increase when moving from very-short (moments and hours) to short (past few days) time frames, and that response times will remain unchanged or even decrease for longer time frames (past weeks and months). This would be represented by a curvilinear relationship or a “piecewise linear” relationship (with an inflection point indicating the shift from episodic to semantic retrieval) between the time frames and response times.

#### Response levels

Following similar assumptions as in the case of the response times, we predicted that the relationship between the time frames and response levels will be best represented by a curvilinear trend as well as by a two-segment piecewise linear model. For recent time frames, moving from very brief (hours) to short (days) reporting periods not only allows for including more instances of the given experience but also should be associated with an increased probability of incorporating more intense experiences [5], leading to the increase in response levels as the time frames increase. If times frames covering weeks and months involve belief-based knowledge, this should result in similar response levels regardless of the time frame used. This would be indicated by a flat slope for longer time frames covering weeks and months.

## Materials and methods

### Participants

The sample consisted of 519 adults recruited via Amazon’s Mechanical Turk (MTurk) website. MTurk is an Internet-based platform which allows to recruit study participants for purposes of performing tasks such as online questionnaire completion. Studies administered through MTurk reach a more diverse population than many convenience samples [19,20] and produce high-quality and reliable data equivalent to data collected using more traditional methods [20–24]. The study was limited to individuals aged 18 years and over who were located in the United States, fluent in English, and with high approval ratings in previous MTurk studies (> 90%). These eligibility criteria were based on the recent recommendations in the field of crowdsourcing methods [19,22,24,25]. Participants were paid $0.80 for completing the 15-minute study. Respondents who did not pass one or more quality check questions (*n* = 43, 8.3%, described below) or reported that they did not understand the instructions (*n* = 7, 1.5%) were excluded from the analyses, resulting in a final sample of 469 participants. The demographics of the sample are reported in Table 1. The Institutional Review Board at the University of Southern California—University Park Campus approved this study.

**Table 1:** Demographic characteristics of the sample (*N* = 469).

Demographics	Percentage
Age^a^	34.7 (11.1; 18–70)
Female	46.1
Race/ethnicity	
African American	5.8
Asian	7.7
Native American	0.9
White	82.7
Other	0.6
More than One Race	2.4
Marital status	
Never married	44.6
Married	33.9
Living with partner	13.7
Divorced	7.0
Widowed	0.9
Currently employed	80.4
Education	
8^th^ to 11^th^ grade	0.2
high school graduate	11.1
some college	31.8
college graduate	46.7
master’s degree	8.1
doctoral degree	2.1
Household income	
Less than $20,000	13.4
$20,000 to $34,999	22.4
$35,000 to $49,999	18.6
$50,000 to $74,999	22.2
More than $75,000	23.5

^a^ *M* (*SD*; range).

### Measures

#### Judgment task

Participants reported the extent to which they experienced 8 states (*excited*, *happy*, *calm*, *sad*, *anxious*, *angry*, *pain*, *stress*) over 12 time frames (*now*, *last 2 hours*, *last 24 hours*, *last 2 days*, *last 3 days*, *last week*, *last 2 weeks*, *last month*, *last 3 months*, *last 6 months*, *last year*, *in general*). Ratings were provided on a 5-point scale (1 = *not at all*; 2 = *a little*; 3 = *moderately*; 4 = *quite a bit*; 5 = *extremely*). Each state was crossed with each time frame (96 trials). To reduce participants’ burden and to keep the number of trials similar to the study of Robinson and Clore [8], 59 (61%) of the possible 96 trials were randomly selected for each participant and these were presented to participants in randomized order.

**Fig 1:**
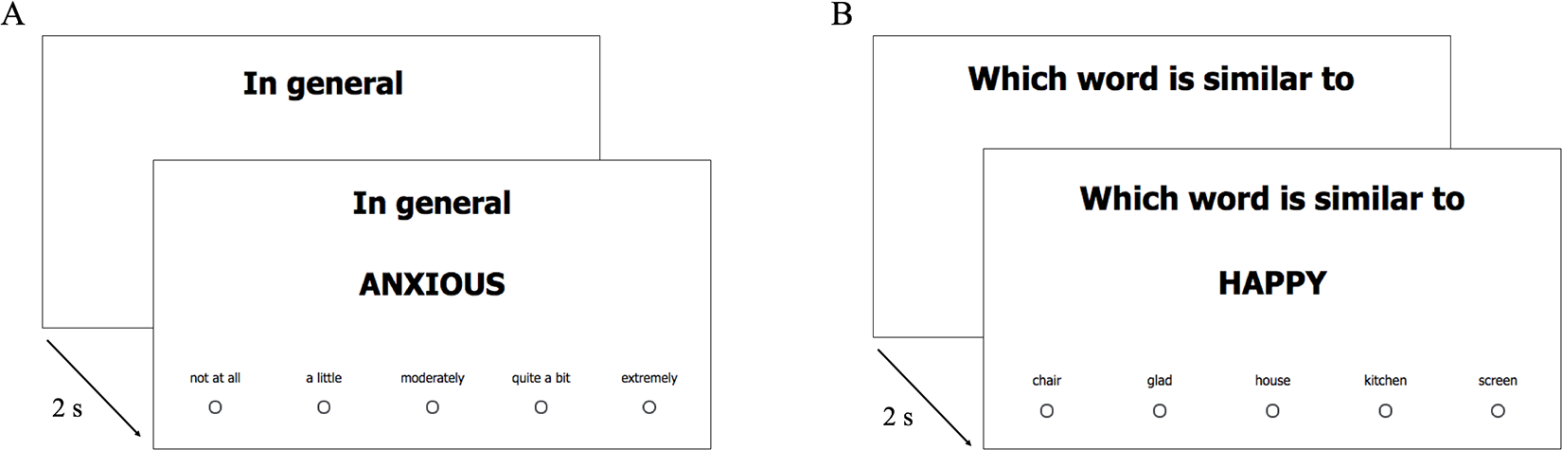
Study procedure. Panel A shows an example of the judgment task trial. Panel B shows an example of the quality check question.

The task was programmed in Qualtrics. The sequence of each judgment trial (see Fig 1A) was as follows. First, the time frame appeared in the center of the screen. After 2 seconds, the state was shown below the time frame together with the response options. Participants selected the response by clicking on a button presented below the response option. Once the response was given, a new trial began automatically. The response time (RT) between the presentation of the state and the response selection was recorded electronically. Participants were asked for quick but accurate responses.

#### Quality check questions

The validity of the findings from this study may be threatened if some participants provided inattentive or careless responses [26,27]. This is of particular concern given the repetitive nature of the task. Thus, three questions were included in different parts of the judgment task to check the quality of the data. Those questions had the same format as the other trials (see Fig 1B), but instead asked for a word similar to *happy*, *sad*, and *anxious*. Out of 5 response choices, the correct one was a synonym (*glad*, *unhappy*, and *worried*, respectively), whereas incorrect choices represented clearly unrelated words (e.g., table, kitchen, fridge).

At the end of the survey, participants were asked whether they understood the instructions with the response options *completely*, *mostly*, *not really* (participants indicating that they did “not really” understand the task were eliminated from the analyses).

### Procedure

Upon accepting the study on MTurk, participants were redirected to the online survey programmed using Qualtrics, a Web-based survey software. First, participants provided informed consent by clicking “next” and completed demographic questions. Then, the instructions for the judgment task were given together with 3 practice trials. The 59 trials of the judgment task were presented in random order. The attention check questions were displayed after the 10th, 34th, and 53rd trial. See S1 File for the screenshots including the judgment task instructions and the examples of a trial and attention check question. Two self-paced breaks were inserted after the 20th (*Mdn* = 7.7 s) and 39th trial (*Mdn* = 5.5 s). During the breaks, participants were asked to relax, refocus their attention on the task, and proceed when ready. After the 27th and 46th trial participants responded to an open question about the reasons why they made this rating (data not reported). The study ended with two personality trait questionnaires, the Life Orientation Test-Revised [28] and the Big Five Inventory-S [29] (data not reported), and the end of survey question. The study took approximately 15 minutes to complete.

### Statistical analyses

#### Data preprocessing

The results from 27,671 trials were inspected for errors in trial presentation and implausible RTs. First, an examination of the trial upload times showed that some trials were not presented as originally programmed, that is, 2 seconds after the time frame presentation. To clean the data, we set an error margin at 10% and removed 802 trials that were presented more than 0.2 seconds earlier or later than programmed. Second, 107 trials yielded implausible RTs (i.e., negative RT values and RTs equal to 0 seconds) and these were removed. Third, some RTs were extremely long (e.g., 600 s or more), which could suggest that participants took a break from the survey during that trial. Such extreme responses (267 trials) were trimmed at the 99^th^ percentile (17 s). For the trials described above, both RTs and ratings were removed, and 26,495 trials with RTs and intensity ratings (95.8%) were retained. Finally, we performed a log transformation of response times to normalize the distribution (results were similar when using untransformed or transformed RT data).

#### Hypotheses testing

*Main analyses*. The hypotheses regarding curvilinear patterns of change in RTs and response levels over different time frames were tested separately for each state in a series of multilevel polynomial growth models. In all models, time frame was included as a continuous Level-1 predictor coded from 0 = *right now* to 11 = *last year*, allowing for random effects (i.e., individual differences) in intercepts and time frame slopes. Following procedures described by Singer and Willet [30], three nested growth models were compared: a no change model, a model assuming linear change, and a model assuming quadratic change (i.e., the hypothesized model). The best fitting model for each outcome variable was determined with likelihood ratio tests comparing change in the deviance statistic (i.e., –2 log-likelihood) between nested models.

*Piecewise regression analyses*. In supplementary models, we tested a series of piecewise linear (i.e., spline) models for each state as an alternative strategy to examine the form of the relationship between RTs/response levels and time frames. If RTs and/or response levels increase for shorter time frames but remain constant (or decrease) for longer time frames (i.e., after the theorized shift from episodic to semantic memory has been reached), one might expect that changes in RTs/response levels would be well represented by a piecewise linear model with two segments. The inflection point separating the two segments is not known. Based on results from Robinson and Clore’s [8] prior research, we first estimated a model using an inflection point at *two weeks*, resulting in two segments (*right now* to *last week* and *last 2 weeks* to *last year*). Linear coefficients for both segments were estimated and compared. To explore whether this “knot” results in the best fitting model, similar piecewise growth models were estimated for other possible knots (*last 24 hours*, *last 2 days*, *last 3 days*, *last week*, *last month*, *last 3 months*, *last 6 months*). Because these models are not nested, we compared the models based on the Bayesian Information Criterion (BIC) to determine which inflection point provides the best fit to the data. Smaller BIC values indicate a better-fitting model. A BIC difference of >10 represents strong evidence for meaningful differences between the models, while >100 represents decisive evidence [31].

*Time frame “in general”*. The time frame *in general* was excluded from the main analyses, because in contrast to the other time frames (*right now*–*last year*) it is not easily quantifiable and may not be an exceptional instance of an extended time frame. To explore whether RTs/response levels to the time frame *in general* differ from the longest time frame *last year*, we compared the mean RTs/response levels between the *in general* and *last year* time frames. *P*-values were adjusted for multiple comparisons using the Benjamini-Hochberg [32] adaptive step-up Bonferroni method.

All analyses were performed with M*plus* Version 8. Missing values (due to participants receiving only a random selection of 59 out of 96 possible trials; participants were not allowed to skip trials) were accommodated using maximum likelihood parameter estimation.

## Results

The dataset is publicly available via Open Science Framework and can be accessed at osf.io/2hkyn.

### Time frame effects on response times

**Fig 2:**
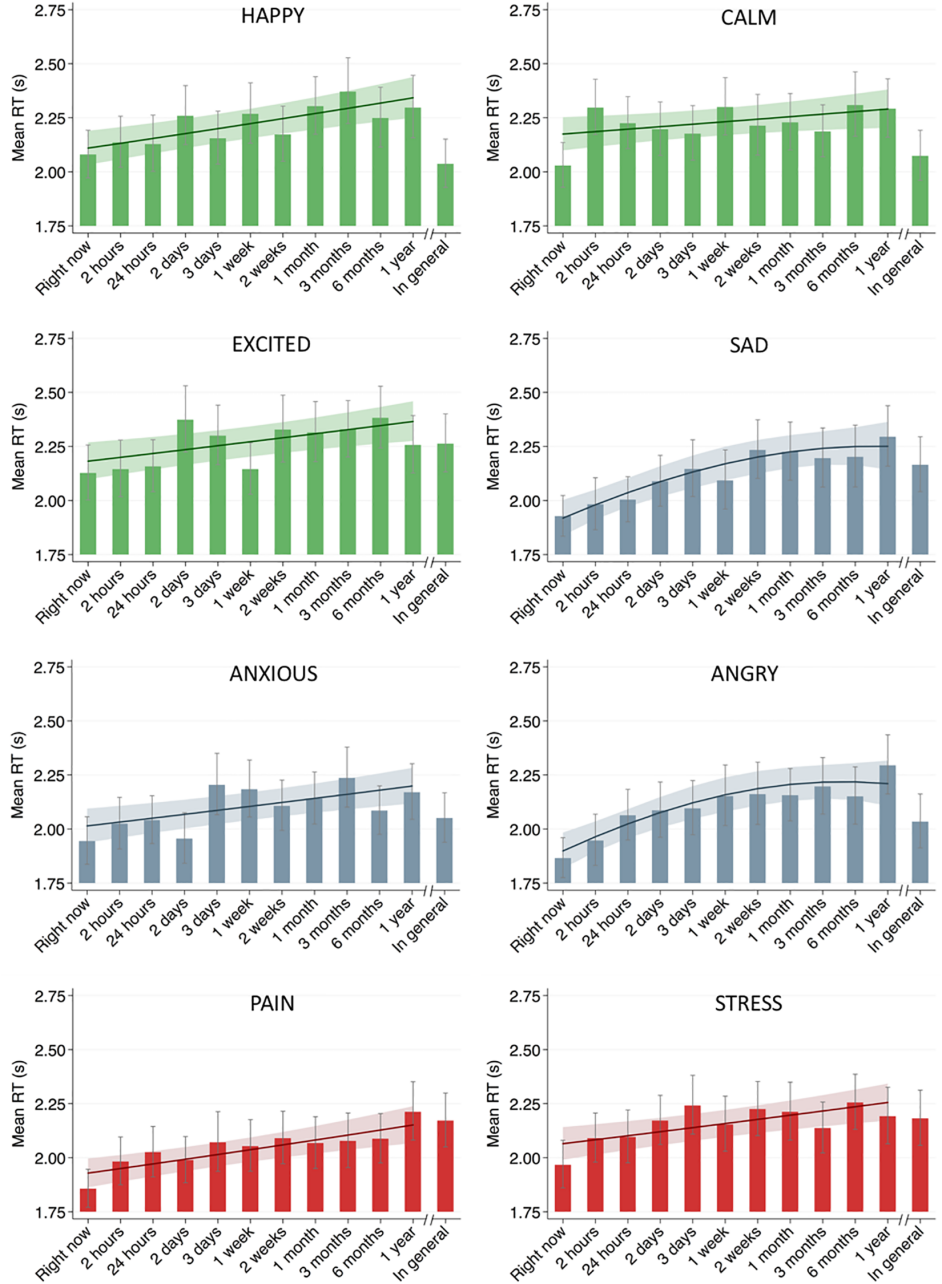
Effect of time frames on response times. Response times (RT) are reported in seconds. Each bar represents mean response time in a given time frame. Each curve represents the change trajectory estimated from the best fitting model for each state. Whiskers and shaded area represent 95% confidence interval.

*Main analyses*. The effects of time frames on response times are illustrated in Fig 2, which includes both the observed means for the individual time frames and the estimated (linear or curvilinear) trends for the best fitting model for each state. For all states, there was a significant and positive relationship between the length of the time frame and RT. The hypothesized model assuming curvilinear change showed the best fit only for two emotions, sad and angry. A linear increase in RTs was evident for the remaining states: happy, calm, excited, anxious, pain, and stress. The detailed comparison of the multilevel polynomial growth models for each state can be found in S1 Table.

*Piecewise regression analyses*. The form of the relation between RTs and time frames was further examined with spline analyses including two segments (Table 2). First, we estimated a model using an inflection point at two weeks. The inspection of linear coefficients for both segments showed that for shorter time frames (from *right now* to *last week*) there was a positive relationship between time frames and RT, whereas for longer time frames (from *last two weeks* to *last year*) this relation was not significant. All states except for calm showed this pattern of results. However, the comparison of the regression coefficients between the two segments indicated that the slopes were significantly different from each other only in the case of angry.

**Table 2:** Comparison of pairwise linear models for response times and response levels data.

	Response times			Response levels		
Knot at:	Segment 1 *M*(*SE*)	Segment 2 *M*(*SE*)	BIC	Segment 1 *M*(*SE*)	Segment 2 *M*(*SE*)	BIC
*Happy*						
24 hours	0.031 (0.035)	0.009 (0.003)**	3964	**0.044 (0.038)**	0.014 (0.005)**	6440
2 days	**0.021 (0.017)**	0.009 (0.004)*	3963	0.045 (0.020)*	0.011 (0.005)*	6435
3 days	0.018 (0.011)	0.008 (0.004)	3963	0.035 (0.013)**	**0.009 (0.006)**	6433
1 week	0.013 (0.008)	0.009 (0.005)	3964	0.028 (0.010)**	**0.008 (0.007)**	6434
2 weeks	0.012 (0.006)*	0.008 (0.006)	3964	0.023 (0.008)**	**0.007 (0.008)**	6437
1 month	0.012 (0.005)*	0.008 (0.008)	3964	0.020 (0.007)**	**0.006 (0.010)**	6436
3 months	0.012 (0.004)**	0.004 (0.011)	3964	0.019 (0.006)**	**0.003 (0.013)**	6437
6 months	0.012 (0.004)**	0.000 (0.017)	3962	**0.018 (0.005)**	**-0.007 (0.019)**	6434
*Calm*						
24 hours	0.088 (0.035)*	**0.001 (0.003)**	3901	-0.251 (0.043)***	-0.025 (0.005)***	6796
2 days	0.032 (0.018)	0.001 (0.004)	3906	-0.155 (0.023)***	-0.017 (0.006)**	6780
3 days	0.017 (0.011)	0.002 (0.004)	3907	-0.108 (0.015)***	**-0.012 (0.006)**	6780
1 week	0.012 (0.008)	0.001 (0.005)	3908	-0.086 (0.011)***	**-0.005 (0.007)**	6780
2 weeks	0.010 (0.006)	0.000 (0.006)	3908	-0.068 (0.009)***	**-0.001 (0.009)**	6790
1 month	0.007 (0.005)	0.002 (0.008)	3908	-0.054 (0.008)***	**-0.001 (0.011)**	6802
3 months	0.005 (0.004)	0.005 (0.011)	3910	-0.046 (0.007)***	**0.004 (0.014)**	6810
6 months	0.005 (0.003)	0.009 (0.018)	3909	-0.041 (0.006)***	**0.010 (0.022)**	6815
*Excited*						
24 hours	0.032 (0.037)	0.007 (0.003)*	3945	0.131 (0.043)**	0.044 (0.006)***	6807
2 days	0.029 (0.017)	0.005 (0.003)	3945	0.114 (0.021)***	0.039 (0.006)***	6798
3 days	0.026 (0.011)*	0.003 (0.004)	3943	0.091 (0.015)***	0.035 (0.007)***	6791
1 week	0.018 (0.008)*	0.002 (0.005)	3942	0.076 (0.011)***	0.033 (0.008)***	6783
2 weeks	0.014 (0.007)*	0.002 (0.006)	3940	0.068 (0.009)***	0.029 (0.010)**	6779
1 month	0.014 (0.006)*	-0.002 (0.008)	3936	0.061 (0.008)***	0.025 (0.012)*	6785
3 months	0.012 (0.004)**	-0.004 (0.011)	3941	0.058 (0.007)***	**0.017 (0.016)**	6792
6 months	0.011 (0.004)**	-0.009 (0.017)	3945	0.052 (0.006)***	0.016 (0.024)	6802
*Sad*						
24 hours	0.035 (0.039)	0.015 (0.004)***	4087	0.046 (0.036)	0.079 (0.005)***	6037
2 days	0.033 (0.018)	0.013 (0.004)**	4088	0.067 (0.018)***	0.079 (0.006)***	6014
3 days	0.032 (0.011)**	0.011 (0.005)*	4083	0.065 (0.012)***	0.081 (0.006)***	6001
1 week	0.029 (0.008)***	0.008 (0.006)	4076	0.06 (0.009)***	0.087 (0.008)***	5994
2 weeks	0.026 (0.006)***	0.006 (0.007)	4074	0.059 (0.008)***	0.096 (0.009)***	5977
1 month	0.024 (0.005)***	0.001 (0.009)	4074	0.061 (0.007)***	0.108 (0.012)***	5943
3 months	0.021 (0.005)***	-0.001 (0.012)	4077	0.065 (0.006)***	0.122 (0.016)***	5931
6 months	0.018 (0.004)***	-0.001 (0.020)	4082	0.068 (0.005)***	0.143 (0.024)***	5953
*Anxious*						
24 hours	0.036 (0.034)	0.008 (0.003)*	3827	0.187 (0.044)***	0.075 (0.006)***	6983
2 days	0.026 (0.017)	0.006 (0.003)	3828	0.183 (0.023)***	0.066 (0.006)***	6951
3 days	0.021 (0.011)	0.005(0.004)	3827	0.140 (0.015)***	0.062 (0.007)***	6949
1 week	0.021 (0.008)**	0.001 (0.005)	3823	0.117 (0.011)***	0.060 (0.008)***	6952
2 weeks	0.018 (0.006)**	-0.001 (0.006)	3825	0.104 (0.009)***	0.058 (0.010)***	6951
1 month	0.015 (0.005)**	-0.001 (0.007)	3827	0.094 (0.008)***	0.057 (0.012)***	6961
3 months	0.013 (0.004)**	-0.004 (0.010)	3828	0.090 (0.007)***	0.052 (0.016)**	6965
6 months	0.012 (0.004)**	-0.014 (0.016)	3827	0.086 (0.006)***	0.049 (0.026)	6963
*Angry*						
24 hours	0.089 (0.040)*	0.012 (0.003)***	4074	0.062 (0.039)	0.068 (0.005)***	5681
2 days	0.056 (0.018)**	0.009 (0.004)*	4078	0.081 (0.021)***	0.066 (0.006)***	5636
3 days	0.039 (0.011)***	**0.008 (0.004)**	4081	0.072 (0.014)***	0.066 (0.006)***	5624
1 week	0.032 (0.008)***	**0.006 (0.005)**	4082	0.065 (0.010)***	0.069 (0.008)***	5623
2 weeks	0.028 (0.006)***	**0.003 (0.006)**	4082	0.063 (0.008)***	0.071 (0.009)***	5624
1 month	0.023 (0.005)***	0.001 (0.008)	4084	0.063 (0.007)***	0.074 (0.011)***	5638
3 months	0.020 (0.004)***	0.001 (0.011)	4089	0.065 (0.006)***	0.076 (0.015)***	5652
6 months	0.017 (0.004)***	0.001 (0.016)	4091	0.064 (0.005)***	0.094 (0.022)***	5660
*Pain*						
24 hours	0.067 (0.040)	0.008 (0.003)*	3869	0.110 (0.034)**	0.047 (0.005)***	5617
2 days	0.033 (0.018)	0.008 (0.004)*	3871	0.095 (0.017)***	0.043 (0.006)***	5596
3 days	0.021 (0.011)	0.008 (0.004)	3875	0.068 (0.012)***	0.044 (0.006)***	5595
1 week	0.016 (0.008)*	0.008 (0.005)	3874	0.054 (0.009)***	0.047 (0.007)***	5588
2 weeks	0.013 (0.006)*	0.009 (0.006)	3877	0.048 (0.007)***	0.052 (0.009)***	5578
1 month	0.012 (0.005)*	0.010 (0.008)	3879	0.047 (0.006)***	0.055 (0.011)***	5565
3 months	0.011 (0.004)**	0.012 (0.011)	3878	0.047 (0.006)***	0.059 (0.015)***	5554
6 months	0.010 (0.004)**	0.017 (0.017)	3879	0.046 (0.005)***	0.077 (0.023)**	5537
*Stressed*						
24 hours	0.084 (0.038)*	0.005 (0.003)	3784	0.072 (0.045)	0.100 (0.006)***	7260
2 days	0.045 (0.017)**	0.003 (0.003)	3784	0.131 (0.023)***	0.094 (0.007)***	7243
3 days	0.034 (0.010)**	0.001 (0.004)	3782	0.116 (0.015)***	0.093 (0.008)***	7241
1 week	0.026 (0.007)**	-0.001 (0.005)	3782	0.108 (0.012)***	0.093 (0.009)***	7239
2 weeks	0.019 (0.006)**	-0.002 (0.006)	3784	0.107 (0.010)***	0.090 (0.011)***	7232
1 month	0.015 (0.005)**	-0.003 (0.007)	3785	0.105 (0.008)***	0.087 (0.014)***	7227
3 months	0.013 (0.004)**	-0.005 (0.010)	3786	0.103 (0.007)***	0.085 (0.018)***	7224
6 months	0.011 (0.003)**	-0.005 (0.016)	3787	0.100 (0.007)***	0.086 (0.028)**	7226

*Note*. Models were estimated for all possible knots (*last 24 hours*, *last 2 days*, *last 3 days*, *last week*, *last 2 weeks*, *last month*, *last 3 months*, *last 6 months*). Linear coefficients and standard errors for corresponding segments and Bayesian Information Criterion (BIC) are displayed. Segment 1 includes time frames before the knot. Segment 2 includes the knot and time frames after the knot including the *last year* time frame.

The difference between pairs of coefficients printed in bold is significant at *p* < .05.

* *p* < .05.

** *p* < .01.

*** *p* < .001.

To explore whether this inflection point leads to the best fitting model, we estimated spline models for all possible knots and compared the BIC indices of the models. As displayed in Table 2, a comparable pattern of results, that is, a linear increase for shorter time frames and a lack of association for longer time frames, was present for almost all knots longer than *last week*. However, with very few exceptions, the regression slopes for shorter and longer time frames were not significantly different from each other. Model comparison based on the BIC showed only very small differences in BIC values, providing no indication that a specific inflection point would be preferable to yield the best fitting model.

### Time frame effects on response levels

**Fig 3:**
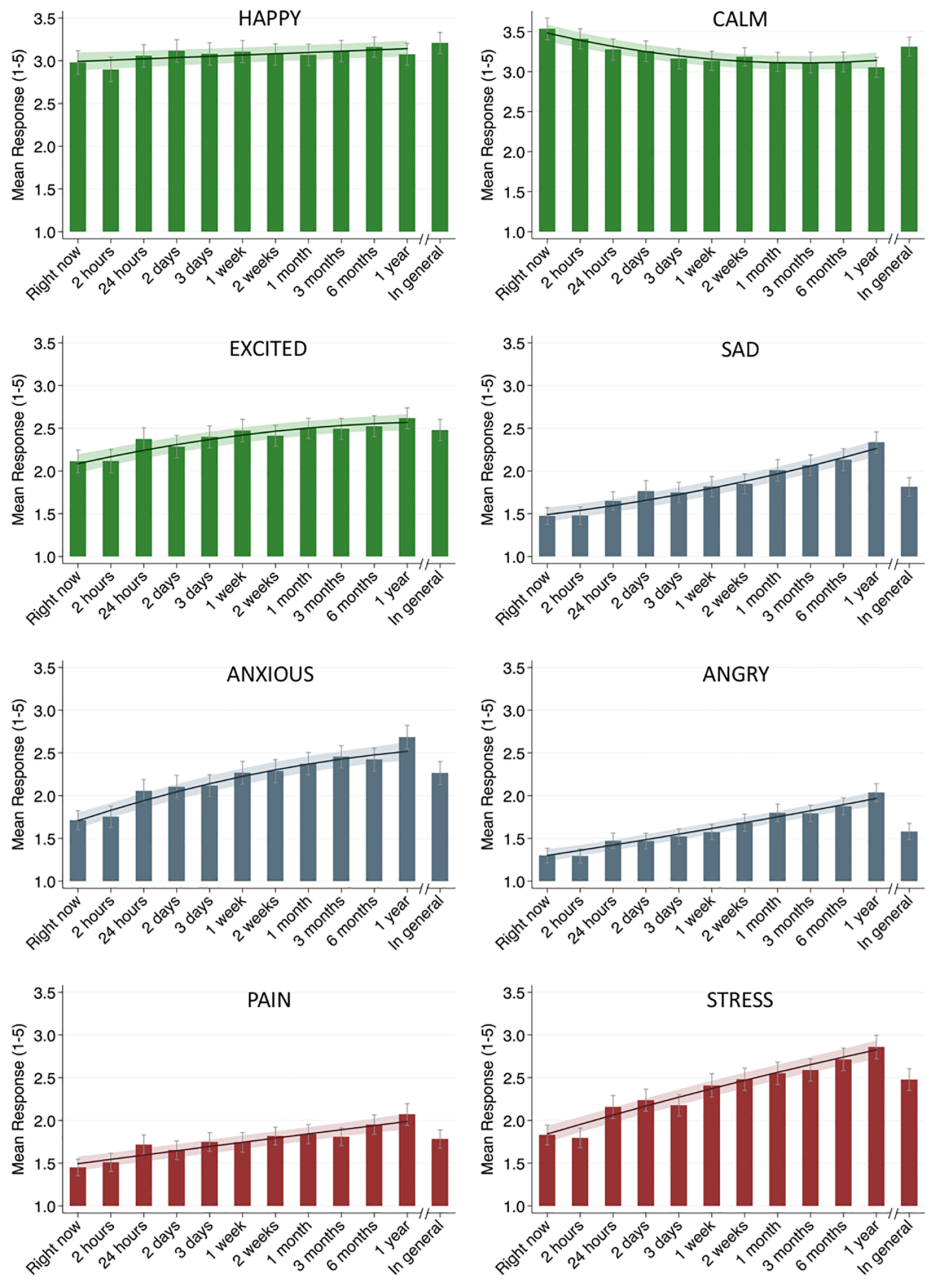
Effect of time frames on response levels (1–5). Each bar represents mean response level in a given time frame. Each curve represents the change trajectory estimated from the best fitting model for each state. Whiskers and shaded area represent 95% confidence interval.

*Main analyses*. Fig 3 displays the effects of time frames on response levels for each state. Longer time frames were associated with higher ratings, as indicated by a positive relationship between the length of time frame and response. This positive association was significant for all states except for calm, which showed a reverse association, such that longer time frames were related to significantly lower ratings. For the majority of states, a model assuming curvilinear change fitted the data significantly better than the model assuming linear change. However, the nature of the curvilinear trajectories differed between the states. The hypothesized flattening of the slope, as indicated by a negative quadratic term, was observed for the states excited, anxious, pain, and stressed. Contrary to hypotheses, the quadratic term for states sad and angry was positive, indicating that the rate of increase in response levels became more (rather than less) pronounced for longer time frames. A linear increase in response levels was evident for the state happy. Detailed information concerning the multilevel polynomial growth models for each state is shown in S2 Table.

*Piecewise regression analyses*. The form of the relation between response levels and time frames was further examined with spline analyses (Table 2). First, linear coefficients for the model using the inflection point of *two weeks* were compared. An increase in response levels was observed in segments covering both shorter and longer time frames, with the exception of happy and calm, for which the increase of response levels in the segment of longer time frames (2 weeks and longer) was nonsignificant.

Next, spline models for all possible knots were estimated and compared. The pattern observed for the inflection point of *two weeks* appeared for almost all other knots longer than *2 days*–the length of the time frame was positively related to response level for both short and long time frames. Model comparisons based on the BIC did not point to a specific knot resulting in the best fitting model across states.

### Time frame *in general*

The mean RTs and response levels for time frame *in general* are illustrated in Figs 2 and 3, respectively. These figures show that both response times and levels for this time frame might differ from the longest time frame included in the main analyses, *last year*. The test of this difference can be approached in two ways. First, the means for time frame *in general* can be compared with means for *last year*. Second, the means can be compared with the growth model estimated value for the time frame *last year*. The results based on both approaches are comparable (Table 3). With regard to RTs, less time was necessary to answer to the *in general* time frame for states happy, calm, and angry. The differences for other states were not significant. The analysis of the response levels shows that for the majority of states, the responses given to time frame *in general* are significantly lower than the responses concerning *last year*. This was not the case for positive emotions happy and calm, for which ratings for *in general* were either not different from (happy) or higher than (calm) those for the *last year* time frame.

**Table 3:** The comparison of means of response times and response levels for *in general* and *last year* time frames.

	Response times		Response levels	
	Comparison A	Comparison B	Comparison A	Comparison B
Variable	*M*(*SE*)	*M*(*SE*)	*M*(*SE*)	*M*(*SE*)
Happy	-0.14 (0.04)**	-0.15 (0.04)***	0.10 (0.06)^a^	0.06 (0.04)
Calm	-0.10 (0.04)*	-0.09 (0.04)*	0.26 (0.06)***	0.19 (0.05)***
Excited	-0.01 (0.04)	-0.05 (0.04)	-0.17 (0.06)**	-0.13 (0.05)**
Sad	-0.06 (0.04)	-0.04 (0.04)	-0.53 (0.06)***	-0.39 (0.04)***
Anxious	-0.05 (0.04)	-0.06 (0.03)	-0.37 (0.06)***	-0.23 (0.05)***
Angry	-0.11 (0.04)*	-0.09 (0.04)	-0.45 (0.06)***	-0.41 (0.04)***
Pain	-0.02 (0.04)	0.01 (0.04)	-0.29 (0.06)***	-0.23 (0.04)***
Stress	0.00 (0.04)	-0.02 (0.04)	-0.33 (0.07)***	-0.35 (0.06)***

The table presents (A) a paired-samples *t*-test and (B) the comparison of the mean for the *in general* time frame with the growth model estimated value for the *last year* time frame. *P*-values were adjusted for multiple (i.e., eight) comparisons using the Benjamini-Hochberg adaptive step-up Bonferroni method.

^a^ *p* < .10.

* *p* < .05.

** *p* < .01.

*** *p* < .001.

## Discussion

The degree to which episodic and semantic memory processes contribute to retrospective self-reports has been reported to depend on the length of the reporting period. Robinson and Clore [4] suggested that when the amount of accessible detail decreases due to lengthy reporting periods, an episodic retrieval strategy is abandoned in favor of a semantic, belief-based retrieval strategy. The current study further investigated this shift between episodic and semantic retrieval strategies by conceptually replicating the study of Robinson and Clore [8] for both affective and somatic states and by attempting to localize the exact reporting period at which the theorized shift occurs. A relatively large sample of adults rated their emotions and symptoms over a broad range of time frames. We found that the relationship between the time frames and RTs/response levels was similar for emotions and symptoms. However, taking the results of both RTs and response levels into account, we found mixed evidence to support the findings of Robinson and Clore [8]. The main finding was that we observed an increase in both RTs and response levels with longer time frames, but found no consistent evidence for a change in response patterns that would suggest a shift in retrieval strategies for longer time frames.

In line with previous findings [8], the RTs to all cues were relatively short (below 3s), indicating that individuals were on average relatively fast to evaluate their states over different reporting periods. However, they needed more time to respond to the question when it covered a longer reporting period. Robinson and Clore [8] interpreted the pattern of RTs as an indication of different memory retrieval strategies. Following this assumption, the increase in RTs observed in this study suggests that, even for longer time frames, individuals make an effort to retrieve some episodic details about this period instead of entirely abandoning the episodic retrieval in favor of belief-based retrieval only. This implies that the cognitive heuristics, which depend on the access to episodic details, might also influence ratings related to longer time frames.

Turning to response levels, we observed higher ratings for longer time frames for both emotions and symptoms (i.e., more intense emotions and symptoms), consistent with prior findings showing that the reporting period has a pronounced effect on ratings of both emotions [5,8,33] and symptoms [14,15,17]. As with RTs, our finding is in line with the idea that cognitive heuristics impact recall ratings involving both shorter and longer time periods. According to the peak-end rule [34], the retrospective evaluation of an experience tends to be disproportionally affected by the most salient (“peak”) and recent moments. What constitutes a salient or “peak” experience, however, depends on the length of the reporting period, in that longer time frames have a greater probability of incorporating more intense “peak” events. Similarly, when asked to spontaneously describe prototypical experiences happening over extended periods of time (e.g., last year), individuals refer to more severe events than when the question inquires about shorter time frames (e.g., last week) [35]. Thus, people may give higher intensity ratings for longer time frames because they draw on more extreme moments or events that are available or salient in memory. For example, in a study that examined the effect of time frames on the ratings of anger, respondents referred to more serious (trivial vs. major life event) and intense (minor irritation vs. rage) experiences of anger when responding to the longer time frame (year vs. week) [35].

The increase in mean response levels was consistently evident across all states with the exception of calm. The reversed pattern observed for calm might be a consequence of the testing context—the judgment task is a monotonous procedure during which individuals might feel more calm than usual, resulting in higher levels for *right now* than for longer time frames. Additionally, the pattern described above did not extend to the responses to the *in general* time frame. When participants evaluated their negative emotions and symptoms *in general*, they rated them significantly lower compared to the *last year* time frame. This pattern was less consistent for positive emotions with general ratings being higher for calm, lower for excited, and the same for happy. The finding that general ratings of positive emotions tended to remain at rather high levels, while negative emotions and symptoms were reported as less intense, could reflect processes preserving a positive view of the self [36]. Moreover, the differences between the *in general* and *last year* time frames support our assumption that *in general* it is not necessarily an exceptional instance of an extended time frame.

We also highlight that a similar pattern of recall was observed for symptoms and emotions, with increases in both RTs and response levels. This finding has implications for studies investigating associations between affective states and symptoms. It has previously been shown that physiological responses show a stronger association with ambulatory measurements (*right now*) than with trait measures (see [1] for a review). Similarly, using different reporting periods for the measurement of emotions and symptoms could influence the strength of associations between them. In the present study, the correlations between symptoms and emotions differed considerably depending on the time frames selected (the correlations between the two symptoms (pain and stress) and selected emotions (sad, anxious, and stress) across all time frames are presented in S1 Fig). This suggests that the use of different recall periods within and between studies may quite substantially contribute to inconsistent study results.

The fact that our study did not replicate previous findings might be partly explained by a number of differences in study design and analysis. First, we modified the number and wording of time frames used in the judgment task. Previous studies [8,13] used a small number of time frames which included vague descriptors, such as last few hours, last few weeks, or last few years. We decided to select a large variety of clearly defined, quantifiable time frames for two reasons: to detect the possible shift between retrieval strategies with more precision and to facilitate the generalizability of our findings to symptom research, which predominantly uses well-defined reporting periods such as last 24 hours, past week, or past month [7]. Using a vague descriptor such as “few” reduces the specificity of a time frame. This could impact the retrieval strategy, such that even relatively short but vague time frames (e.g., few weeks) could result in predominantly semantic retrieval. On the other hand, when time frames are clearly defined, individuals might attempt to retrieve and aggregate the experiences, even for rather long time frames. This would result in a linear increase in RTs extending to longer time frames such as *last 6 months* or *last year*, as found in the current study. In our study, the only non-quantified time frame was *in general*. The RTs to this time frame were faster in some cases, which could partially support the interpretation that the wording of the reporting periods is responsible for the failure to replicate Robinson and Clore [8]. Future studies should examine whether the reliance on episodic versus semantic memories depends on the vagueness of the time frame as well as its interaction with time frame length.

A second difference was that response time was operationalized as the time from the presentation of the stimulus to the response, whereas the Robinson and Clore [8] study used a two-response procedure in order to dissociate judgment time from rating time. However, it has previously been shown that some participants might engage in judgment processes after indicating that they are ready to provide a response, complicating the interpretation of a two-response procedure [37]. Therefore, we decided to focus on “total” response time (i.e., the time from stimulus presentation to the response). Finally, we adopted a different analytical approach in that we analyzed all states separately instead of aggregating them into positive versus negative emotions and we did not include the *in general* time frame in the analyses regarding the relationship between the time frames and RTs. Had we included *in general* as the longest reporting period, the much faster RTs for this time frame might have disproportionally affected the results and conclusions.

This study has several limitations that could have influenced the results. First, the data were collected online and the technical quality of users’ computers could influence the accuracy of RTs. However, the analyses are based on within-subject comparisons. Thus, even though factors such as computer speed and browser speed may affect the RTs, the results are not influenced by this, assuming that differences in browser speed are predominantly between respondents. Second, technical problems related to the online software could affect the timing of the state presentation. Therefore, the data were inspected for errors and incorrectly displayed trials (3.3%) were excluded from analyses. Third, it is possible that switching between different time frames and states had an impact on the response times on subsequent trials. To minimize carry-over effects, the trials were presented in a random order for each participant. The procedure could potentially be further improved by including either longer inter-trial intervals or an unrelated task between the trials. Finally, the sample consisted of MTurk participants who might be assumed not to be highly attentive and potentially distracted when completing the tasks. However, a recent study has shown that MTurk participants paid more attention to the instructions than college students [38]. To ensure high-quality data, this study was limited to respondent with high approval ratings [25] and included quality check questions which allowed to identify careless respondents (8.3%). Similar proportions of careless responders have been reported in other studies using Internet samples [26,39]. Future studies should address those limitations by replicating this study in other settings, for example in the laboratory using standardized equipment or in the natural environment what could reduce the testing context effect on very brief time frames such as *right now*.

In conclusion, the findings of this study show that time frames have an effect on the self-reported measures of both emotions and symptoms and suggest that episodic retrieval strategy might be used also when evaluations refer to longer time frames. Researchers should be aware of those effects and for a given study should use the same well-defined time frames across all study measures, as results based on heterogeneous reporting periods might not be comparable.

## Supporting information

S1 Fig Correlation between symptoms (pain/stress) and emotions (sad/anxious/happy) for response levels across all time frames. (PDF)

S1 Table Comparison of polynomial growth models for response times data. (PDF)

S2 Table Comparison of polynomial growth models for response levels data. (PDF)

S1 File Screenshots of the judgment task (instructions and an example of a trial) and an example of an attention check question. (PDF)
