# Supplementary material for: The effects of time frames on self-report
Source: PLoS One. 2018 Aug 9;13(8):e0201655. doi: 10.1371/journal.pone.0201655 (PMC6084942; doi:10.1371/journal.pone.0201655)
Supplement: S1 Fig — (PDF) [file pone.0201655.s001.pdf]

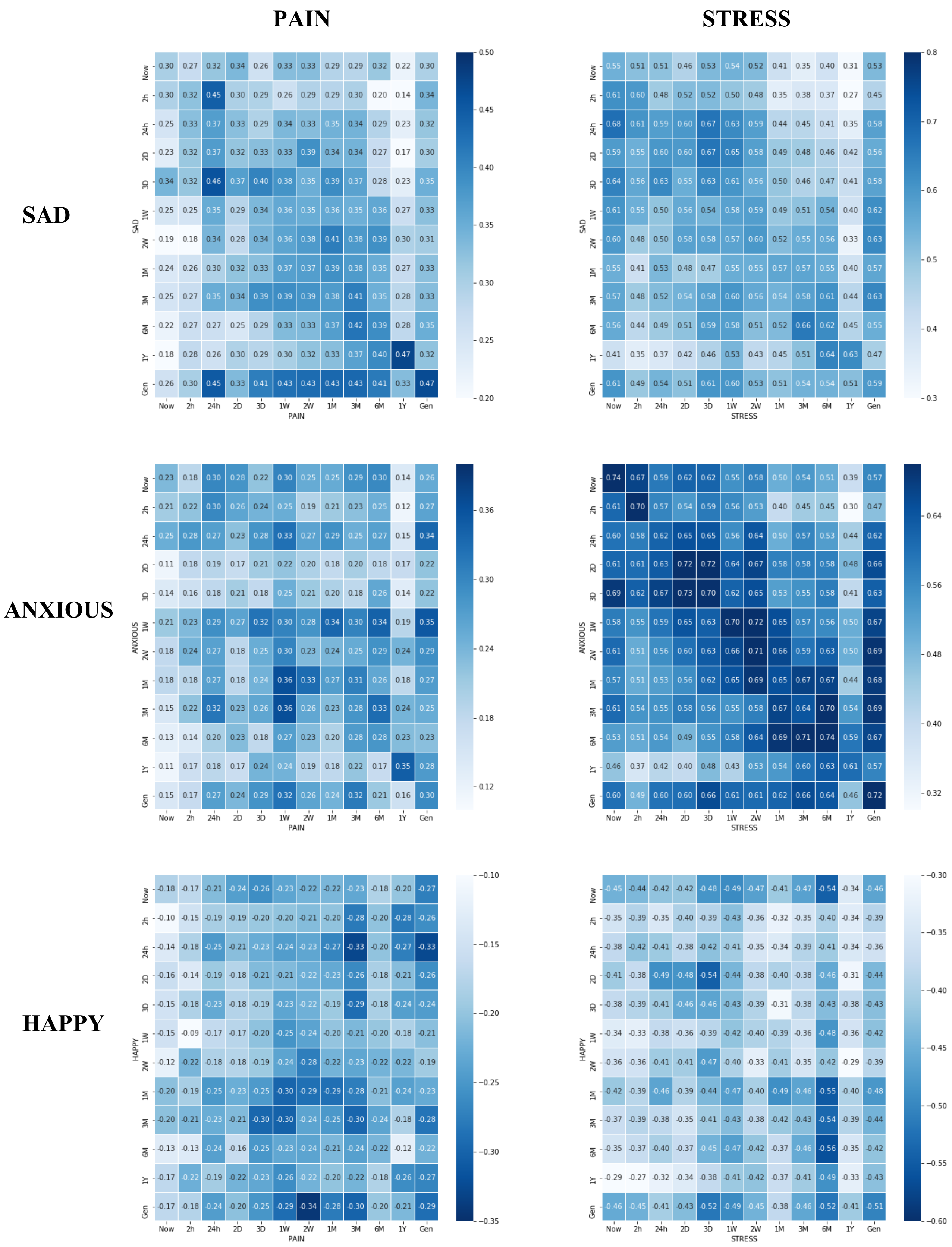

S1 Fig. Correlation between symptoms (pain/stress) and emotions (sad/anxious/happy) for response levels across all time frames.
