# Supplementary material for: The effects of time frames on self-report
Source: PLoS One. 2018 Aug 9;13(8):e0201655. doi: 10.1371/journal.pone.0201655 (PMC6084942; doi:10.1371/journal.pone.0201655)
Supplement: S1 Table — (PDF) [file pone.0201655.s002.pdf]

S1 Table. Comparison of polynomial growth models for response times data. Compared models include no change (Model 1), linear change (Model 2), and quadratic change models (Model 3).

	Happy			Calm		
	Model 1	Model 2	Model 3	Model 1	Model 2	Model 3
Fixed effects:						
Intercept	0.798***	0.747***	0.739***	0.802***	0.776***	0.764***
Time frame (linear)		0.010***	0.016		0.005*	0.014
Time frame^2 (quadratic)			-0.001			-0.001
Variance components:						
Level 1 Residual	0.170***	0.167***	0.167***	0.171***	0.170***	0.169***
Level 2 Intercept	0.086***	0.077***	0.078***	0.068***	0.055***	0.053***
Time frame		0.000	0.001		0.000	0.001
Time frame^2			0.000			0.000
Goodness-of-fit						
BIC	3928	3932	3964	3861	3878	3909
-2*log likelihood	3904	3884	3883	3837	3829	3829
Likelihood ratio test χ^2		19.94***	0.19		7.80[a]	0.90

Note. Covariance components are not reported. χ^2 for comparisons Model 2 vs. Model 1 ($df = 3$) and Model 3 vs. Model 2 ($df = 4$).
[a] $p < .10$, * $p < .05$, ** $p < .01$, *** $p < .001$

	Excited			Sad		
	Model 1	Model 2	Model 3	Model 1	Model 2	Model 3
Fixed effects:						
Intercept	0.820***	0.780***	0.758***	0.756***	0.677***	0.651***
Time frame (linear)		0.008**	0.023*		0.016***	0.033**
Time frame^2 (quadratic)			-0.001			-0.002
Variance components:						
Level 1 Residual	0.170***	0.168***	0.164***	0.182***	0.174***	0.169***
Level 2 Intercept	0.086***	0.090***	0.094***	0.075***	0.074***	0.053***
Time frame		0.000	0.005[a]		0.000**	0.002
Time frame^2			0.000			0.000
Goodness-of-fit						
BIC	3901	3915	3941	4086	4061	4075
-2*log likelihood	3877	3867	3861	4062	4013	3995
Likelihood ratio test χ^2		10.37*	5.82		48.47***	17.94**

Note. Covariance components are not reported. χ^2 for comparisons Model 2 vs. Model 1 ($df = 3$) and Model 3 vs. Model 2 ($df = 4$).
[a] $p < .10$, * $p < .05$, ** $p < .01$, *** $p < .001$

	Anxious			Angry		
	Model 1	Model 2	Model 3	Model 1	Model 2	Model 3
Fixed effects:						
Intercept	0.743***	0.700***	0.677***	0.748***	0.673***	0.640***
Time frame (linear)		0.009**	0.024*		0.015***	0.037**
Time frame^2 (quadratic)			-0.002			-0.002*
Variance components:						
Level 1 Residual	0.160***	0.158***	0.157***	0.179***	0.175***	0.171***
Level 2 Intercept	0.087***	0.095***	0.094***	0.083***	0.073***	0.058***
Time frame		0.000	0.001		0.000	0.002
Time frame^2			0.000			0.000
Goodness-of-fit						
BIC	3788	3798	3826	4077	4061	4082
-2*log likelihood	3764	3750	3746	4053	4013	4002
Likelihood ratio test χ^2		13.92**	3.81		40.41***	11.27*

Note. Covariance components are not reported. χ^2 for comparisons Model 2 vs. Model 1 (*df* = 3) and Model 3 vs. Model 2 (*df* = 4).

^{a} $p < .10$, * $p < .05$, ** $p < .01$, *** $p < .001$

	Pain			Stress		
	Model 1	Model 2	Model 3	Model 1	Model 2	Model 3
Fixed effects:						
Intercept	0.712***	0.657***	0.649***	0.768***	0.725***	0.694***
Time frame (linear)		0.011***	0.016		0.009***	0.029**
Time frame^2 (quadratic)			0.000			-0.002*
Variance components:						
Level 1 Residual	0.174***	0.170***	0.167***	0.162***	0.160***	0.158***
Level 2 Intercept	0.064***	0.047***	0.034**	0.075***	0.071***	0.066***
Time frame		0.000	0.002		0.000	0.001
Time frame^2			0.000			0.000
Goodness-of-fit						
BIC	3856	3850	3875	3749	3758	3784
-2*log likelihood	-3832	-3802	-3795	-3725	-3710	-3704
Likelihood ratio test χ^2		30.61***	6.62		14.74**	6.28

Note. Covariance components are not reported. χ^2 for comparisons Model 2 vs. Model 1 (*df* = 3) and Model 3 vs. Model 2 (*df* = 4).

^{a} $p < .10$, * $p < .05$, ** $p < .01$, *** $p < .001$
