# Supplementary material for: The effects of time frames on self-report
Source: PLoS One. 2018 Aug 9;13(8):e0201655. doi: 10.1371/journal.pone.0201655 (PMC6084942; doi:10.1371/journal.pone.0201655)
Supplement: S2 Table — (PDF) [file pone.0201655.s003.pdf]

S2 Table. Comparison of polynomial growth models for response levels data. Compared models include no change (Model 1), linear change (Model 2), and quadratic change models (Model 3).

	Happy			Calm		
	Model 1	Model 2	Model 3	Model 1	Model 2	Model 3
Fixed effects:						
Intercept	3.065***	2.992***	2.966***	3.217***	3.388***	3.481***
Time frame (linear)		0.015**	0.032*		-0.035***	-0.096***
Time frame^2 (quadratic)			-0.002			0.006***
Variance components:						
Level 1 Residual	0.317***	0.269***	0.263***	0.391***	0.316***	0.301***
Level 2 Intercept	0.895***	1.144***	1.171***	0.756***	0.992***	1.023***
Time frame		0.004***	0.013***		0.006***	0.021**
Time frame^2			0.000***			0.000*
Goodness-of-fit						
BIC	6524	6410	6436	6987	6791	6789
-2*log likelihood	6500	6361	6356	6963	6742	6709
Likelihood ratio test χ^2		138.85***	5.25		220.65***	33.67***

Note. Covariance components are not reported. χ^2 for comparisons Model 2 vs. Model 1 (df = 3) and Model 3 vs. Model 2 (df = 4).
* $p < .05$, ** $p < .01$, *** $p < .001$

	Excited			Sad		
	Model 1	Model 2	Model 3	Model 1	Model 2	Model 3
Fixed effects:						
Intercept	2.381***	2.145***	2.084***	1.835***	1.446***	1.494***
Time frame (linear)		0.048***	0.085***		0.077***	0.045***
Time frame^2 (quadratic)			-0.004*			0.003*
Variance components:						
Level 1 Residual	0.421***	0.314***	0.289***	0.357***	0.233***	0.206***
Level 2 Intercept	0.758***	1.143***	1.080***	0.672***	0.707***	0.577***
Time frame		0.007***	0.029***		0.005***	0.025***
Time frame^2			0.000***			0.000***
Goodness-of-fit						
BIC	7138	6791	6782	6737	6037	5965
-2*log likelihood	7114	6743	6702	-6713	-5989	-5884
Likelihood ratio test χ^2		370.88***	41.72***		724.46***	104.30***

Note. Covariance components are not reported. χ^2 for comparisons Model 2 vs. Model 1 (df = 3) and Model 3 vs. Model 2 (df = 4).
* $p < .05$, ** $p < .01$, *** $p < .001$

	Anxious			Angry		
	Model 1	Model 2	Model 3	Model 1	Model 2	Model 3
Fixed effects:						
Intercept	2.189***	1.782***	1.713***	1.624***	1.286***	1.301***
Time frame (linear)		0.081***	0.126***		0.068***	0.060***
Time frame^2 (quadratic)			-0.004**			0.001
Variance components:						
Level 1 Residual	0.476***	0.329***	0.299***	0.329***	0.225***	0.191***
Level 2 Intercept	0.800***	0.853***	0.695***	0.368***	0.419***	0.378***
Time frame		0.007***	0.034***		0.005***	0.038***
Time frame^2			0.000***			0.000***
Goodness-of-fit						
BIC	7568	6984	6945	6271	5684	5608
-2*log likelihood	-7544	-6936	-6865	6247	-5636	-5528
Likelihood ratio test χ^2		608.15***	71.32***		610.33***	108.15***

Note. Covariance components are not reported. χ^2 for comparisons Model 2 vs. Model 1 ($df = 3$) and Model 3 vs. Model 2 ($df = 4$).

* $p < .05$, ** $p < .01$, *** $p < .001$

	Pain			Stress		
	Model 1	Model 2	Model 3	Model 1	Model 2	Model 3
Fixed effects:						
Intercept	1.751***	1.501***	1.496***	2.358***	1.869***	1.843***
Time frame (linear)		0.049***	0.051***		0.098***	0.114***
Time frame^2 (quadratic)			0.000			-0.002
Variance components:						
Level 1 Residual	0.294***	0.201***	0.178***	0.563***	0.369***	0.338***
Level 2 Intercept	0.604***	0.721***	0.597***	0.750***	0.917***	0.758***
Time frame		0.006***	0.022***		0.008***	0.030***
Time frame^2			0.000***			0.000***
Goodness-of-fit						
BIC	6098	5619	5560	7943	7251	7227
-2*log likelihood	6074	5571	5479	7919	7202	7147
Likelihood ratio test χ^2		503.74***	91.15***		716.45***	55.22***

Note. Covariance components are not reported. χ^2 for comparisons Model 2 vs. Model 1 ($df = 3$) and Model 3 vs. Model 2 ($df = 4$).

* $p < .05$, ** $p < .01$, *** $p < .001$
