# Supplementary material for: The effects of time frames on self-report
Source: PLoS One. 2018 Aug 9;13(8):e0201655. doi: 10.1371/journal.pone.0201655 (PMC6084942; doi:10.1371/journal.pone.0201655)

Instructions for the judgment task (4 pages + 3 practice trials)

INSTRUCTIONS (1/4)

In this study, you will be asked to rate the extent to which you have experienced various feelings across different time frames. You will be asked to rate one feeling at a time.

The response options include: *not at all, a little, moderately, quite a bit, extremely.*

Please select the answer that best describes your experiences.

NEXT

INSTRUCTIONS (2/4)

The sequence of each trial will be as follows:

First, you will see a time frame (e.g., *last month*) in the middle of the screen.

Last month

NEXT

INSTRUCTIONS (3/4)

A few seconds later, a second word (e.g., *relaxed*) will automatically appear under the time frame. At this moment, please determine the extent to which you have experienced the given feeling during the given time frame. In this example: ***relaxed*** during the ***last month***.
The response options are: *not at all, a little, moderately, quite a bit, extremely.*

As soon as you are ready to give your answer, select the response that best describes your experiences during the given time frame. Once you select your answer, it will be registered and the following trial will start automatically. You will not be able to change your response afterward.

Be **accurate**. Do not be in such a hurry to respond that you regret your decision. While being accurate, try to respond as **quickly** as possible.

Last month

RELAXED

not at all	a little	moderately	quite a bit	extremely
○	○	○	○	○

INSTRUCTIONS (4/4)

In addition, you will be sometimes asked to respond to the following question:

Why did you give this rating?

In the space provided, we would like you to describe how you came up with this rating. What were you thinking about as you were deciding how to rate your experience?

Before we begin the actual task, you will complete 3 practice trials.

Click NEXT to begin the practice trials.

NEXT

PRACTICE TRIAL

Last 24 hours

PRACTICE TRIAL

When you are ready to give an answer, select one of the following responses:

Last 24 hours

WORRIED

not at all	a little	moderately	quite a bit	extremely
○	○	○	○	○

PRACTICE TRIAL

In general

PRACTICE TRIAL

When you are ready to give an answer, select one of the following responses:

In general

CONFIDENT

not at all	a little	moderately	quite a bit	extremely
○	○	○	○	○

PRACTICE TRIAL

Last week

PRACTICE TRIAL

When you are ready to give an answer, select one of the following responses:

Last week

CHEERFUL

not at all	a little	moderately	quite a bit	extremely
○	○	○	○	○

Why did you give this rating?

NEXT

Those were the practice trials. Now let's start the task.

There will be 59 trials like the ones you just did. In every trial, you are asked to rate to what extent you have experienced a given feeling during the given time frame.
As soon as you are ready to give an answer, select one of the five responses: *not at all*, *a little*, *moderately*, *quite a bit*, or *extremely*.

Work as accurately and quickly as you can.

NEXT

Example of one trial

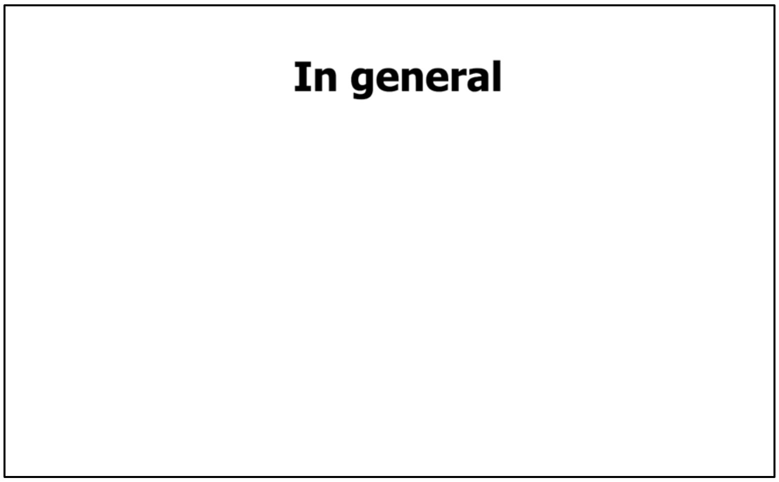

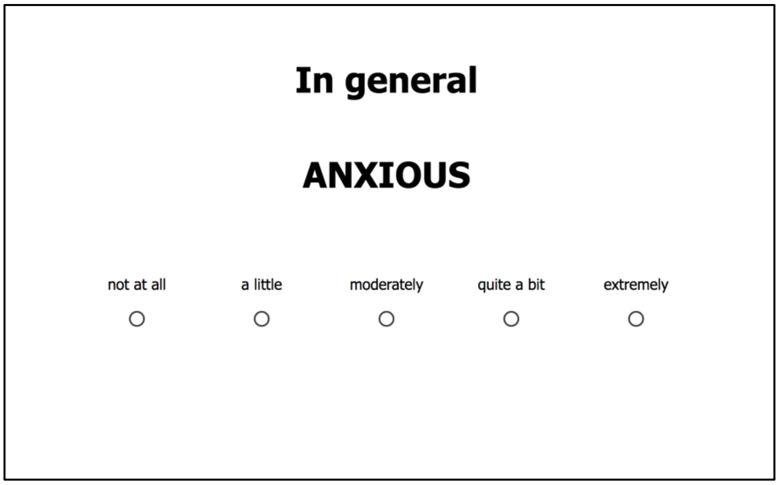

Example of a quality check question

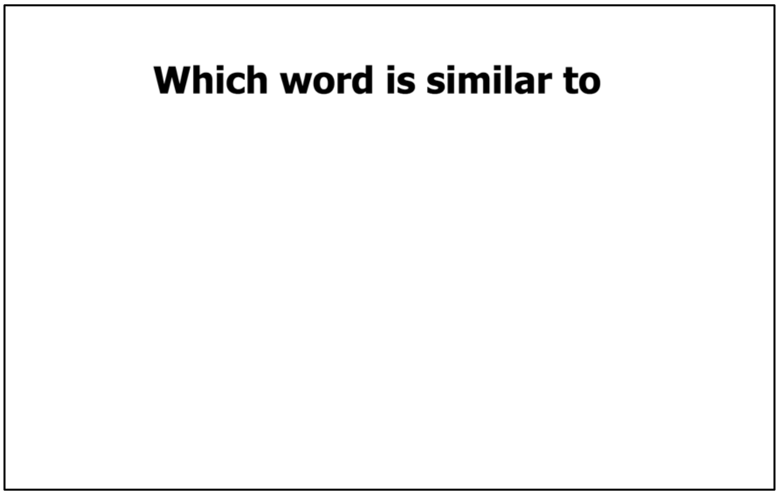

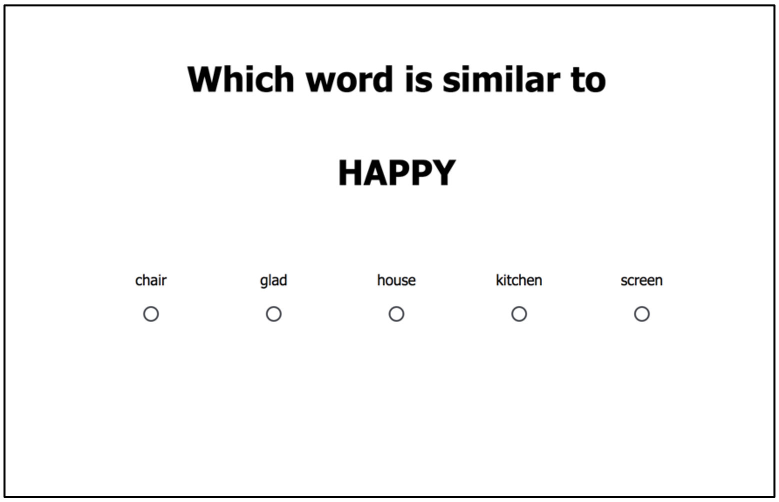

Supplement: S1 File — (PDF) [file pone.0201655.s004.pdf]
